# The effectiveness of video animations in the education of healthcare practitioners and student practitioners: a systematic review of trials

**DOI:** 10.1007/s40037-022-00736-6

**Published:** 2022-12-06

**Authors:** Peter Knapp, Nadia Benhebil, Ella Evans, Thirimon Moe-Byrne

**Affiliations:** 1 Department of Health Sciences & the Hull York Medical School, University of York, York, UK; 2 Hull York Medical School, University of York, York, UK; 3 Department of Health Sciences, University of York, York, UK

**Keywords:** Video, Animation, RCT, Practitioner education

## Abstract

**Introduction:**

Video animations are increasingly available in education but without systematic evaluation. This review aimed to collate trials of animations versus other delivery, in student or qualified healthcare practitioners.

**Methods:**

Included studies had the following features: controlled design with random or quasi-random allocation; student or qualified healthcare practitioners; comparing video animation with another format (e.g. textbook, lecture, static images); animation delivered instead of, or in addition to, another format. The primary outcome was knowledge; secondary outcomes were attitudes and cognitions, and behaviours. Multiple databases were searched from 1996-October 2022 using a defined strategy. We also undertook citation searching. Dual, independent decision-making was used for inclusion assessment, data extraction, and quality appraisal. Included studies were appraised using the Cochrane ROB2 tool. Findings were reported using narrative synthesis.

**Results:**

We included 13 studies: 11 recruited student practitioners, two recruited qualified practitioners, total *n* = 1068. Studies evaluated cartoon animations or 2D/3D animations. Knowledge was assessed in ten studies, showing greater knowledge from animations in eight studies. Attitudes and cognitions were assessed in five studies; animations resulted in positive outcomes in three studies, no difference in one study, and worse outcomes in one study. Behaviours were assessed in three studies, animations producing positive outcomes in two studies and there was no difference in one study. Overall risk of bias was ‘high’ in ten studies and ‘some concerns’ in three.

**Discussion:**

Overall the evidence base is small with mostly ‘high’ risk of bias. Video animations show promise in practitioner education, particularly for effects on knowledge, but bigger, better research is needed.

**Supplementary Information:**

The online version of this article (10.1007/s40037-022-00736-6) contains supplementary material, which is available to authorized users.

## Background

The rapid development of information technologies over recent decades and near-universal access to the internet have revolutionised people’s access to informational and educational resources. These changes have generated opportunities for the use of multimedia in education, including the education of healthcare practitioners, at both pre-registration and post-registration levels. The potential benefits are many: for example, increased student and practitioner choice in the timing and location of content delivery; the potential for individuals to choose a format of delivery that suits their learning style, whether as a general preference or for specific content; and the potential for cost savings. Students’ levels of attention may be enhanced when two channels (e.g. audio and visual) are being stimulated rather than one, as asserted by dual channel theory [1] and which would provide support for the effectiveness of animations. Furthermore, digital provision (such as animations) may enable the effective ‘signalling’ of content, to the benefit of learners [2, 3] and be more efficient in terms of the cognitive load it imposes [4].

Video animations are being developed for use in education as a relatively inexpensive resource. They may be particularly valuable for conveying procedures (such as surgical techniques) or complex content that is more difficult to portray through static images in textbooks or slides (e.g., anatomy or physiological processes) [5]. Animations can use a range of formats, including cartoon portrayal, three-dimensional (3D) or two-dimensional (2D) visualisation, avatars, ‘white board animations’, or a combination. Claims have been made for their effectiveness as informational and educational tools, but the claims are not always based on trial-level evaluations [6]. However, there is trial-level evidence that animations can have positive effects on knowledge when used with patients [7] and a meta-analysis of their effects in non-healthcare education showed a benefit of animations over static images, particularly when the animations were ‘representational’ [8]. There is also some trial-level evidence for the benefits of computer-aided and multimedia methods in science education, but the evidence is not specific to animations [9–11]. Indeed, when trial-level evidence has been generated, findings have not always been positive and may be counter-intuitive. For example, one large panel study in the USA concluded that static images are better than animations for conveying risk information to the public and that animations may lead to confusion [12].

Given the uncertainty around the effectiveness of video animations as informational tools, we undertook a systematic review of the effectiveness of video animations, when compared to other forms of education delivery, on knowledge, attitudes and cognitions, and behaviours among student and qualified healthcare practitioners.

### Methods

The protocol for the systematic review was registered on PROSPERO in February 2021: https://www.crd.york.ac.uk/prospero/display_record.php?RecordID=236330

#### Eligibility criteria

Studies were eligible for inclusion if they used a randomised or quasi-randomised controlled trial design and compared a video animation (or animations) with another form of information (e.g., print, audio recording, ‘talking head’ video, video of an actual procedure), either as an alternative or additional format. We did not apply language restrictions and we included conference abstracts only if sufficient content on methods and results were included. All animation types were eligible, including cartoons, avatars, ‘white board animations’ or animated 2D or 3D models. Animations were eligible if they were part of a multi-component information package and the effect of the animation could be isolated. Participants in the studies were healthcare practitioners or student practitioners, watching the animation as part of an educational programme of study or continuing professional development. The primary outcome was knowledge, and secondary outcomes were attitudes and cognitions (such as satisfaction with information, self-confidence) and behaviours (such as procedural skills or behavioural intentions). We included studies reporting either outcome or difference scores (i.e., those comparing pre- and post-intervention scores). We excluded studies if they were uncontrolled, reported hypothetical scenarios, or the animation was compared with no information intervention.

#### Information sources

Five digital databases were searched up to 9 June 2021 (from January 1996 to search date) and updated on 25 October 2022 (from January 2021 to search date): Medline, Embase, PsychInfo (all Ovid), CINAHL complete (EBESCO Host), Cochrane Library (Wiley). Additional searches were undertaken on Open Grey (Opengrey.eu). The searches were developed by a specialist information scientist from the Centre for Reviews and Dissemination at the University of York (an example search strategy is reported in the figure in the Electronic Supplementary Material; other database searches can be provided on request). The searches were undertaken for two linked systematic reviews (this review and another review assessing video animations for patients [7]). As a result, the total hits are reported jointly for the two reviews until the full article screening stage. We also searched the reference lists of included studies (i.e., backwards citation searching), and undertook forwards citation searching via Google Scholar.

#### Study selection

**Fig. 1:**
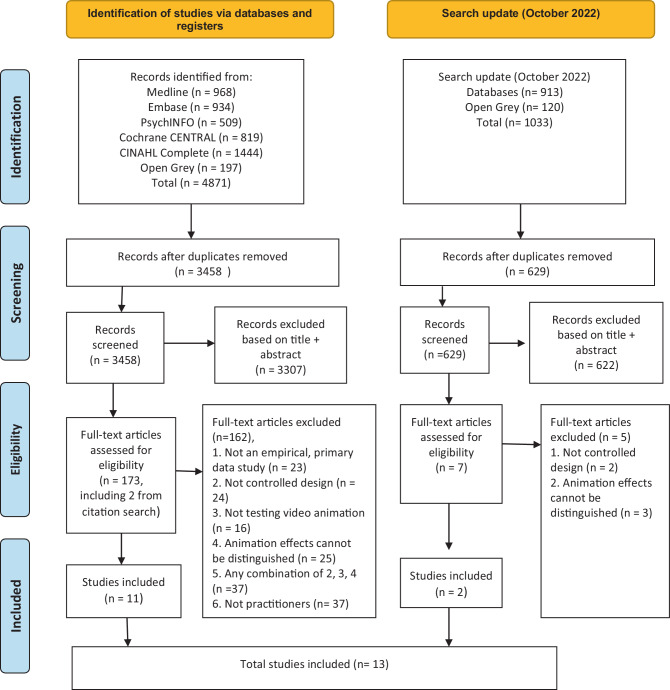
PRISMA flow chart

Search results were exported into EndNote software and de-duplicated. Two researchers (two from EE, NB, PK, TMB) independently screened search results, initially by title and abstract, and then by full text article. Discrepancies were resolved by consensus or by consultation with a third reviewer (PK). See Fig. 1 for PRISMA flowchart.

#### Risk of bias in included studies

We assessed risk of bias using the Cochrane ROB‑2 tool [13], which uses the following five categories: randomisation process; deviation from intended interventions; missing outcome data; outcome measurement; and selection of the reported result. The overall risk of bias rating is derived from the five individual ratings and determined by the ROB2 algorithm. Quality appraisal was undertaken by one researcher (NB, TMB or PK) and checked by a second reviewer.

#### Data extraction and synthesis

Data extraction was undertaken using a standardised data extraction form, which was piloted and refined. It was undertaken by one researcher (NB, TMB or PK) and checked by a second reviewer. When a link to the tested animation was not included in the article, we requested the animation from the corresponding author. The included studies were highly variable (across all Patient-Intervention-Comparator-Outcome or PICO elements) and so the findings have been reported using a narrative synthesis rather than statistical meta-analysis.

## Results

### Study characteristics

The database search (for the two linked systematic reviews) generated 3458 unique hits (Fig. 1). Sifting resulted in 11 eligible studies and an additional two studies were included following citation searches, generating a total of 13 included studies.

Study sample sizes ranged from 22 to 239 (median 60); in total the included trials recruited 1068 participants. Studies had been undertaken in six different countries. All 13 studies were reported in English language journals. Eleven studies were undertaken with student practitioners and only two with qualified practitioners.

Animation topic and style varied greatly. Cartoon animations were used in six studies to show: cardiopulmonary resuscitation (CPR) [14]; first aid [15]; eye surgery [16]; dengue fever transmission [17]; cystoscopy [18]; and the third stage of labour [19]. 3D animated models were used in seven studies on: jaw surgery [6]; DNA replication [20]; oral health [21]; respiratory system [22]; facial transplantation[23]; home safety hazards and assessment in people with dementia [24]; and laparoscopic surgery [25]. No studies featured avatars or ‘white board animations’. Animations lasted 4–10 min, although in six cases the length was not reported. Only four articles had a link to the tested animation.

In five trials the animation was provided in addition to the control intervention: i.e., both groups had a classroom discussion on CPR [14]; both groups saw a video of the surgeon’s view of the procedure [16]; both groups had classroom teaching [19]; both groups also saw a cadaveric video in two of the four trial arms [25]; and both groups received traditional spoken teaching [22].

In nine trials the animations were provided instead of the control intervention, which were: spoken information and a demonstration [15]; either a ‘journalistic’ or ‘dramatic’ style video [17]; spoken information and diagrams [18]; written textbook [6, 20]; 2D images [21]; written text plus static images [23]; static graphics [24] and textbook-only or cadaveric video-only in two of the four trial arms [25].

Participants’ access to animations varied: in seven studies they viewed it only once [6, 16, 17, 19, 21–23], and they viewed it exactly four times in one study [15]. In one study they were asked to view it at least twice [25]. In one study viewing was unlimited [24] and in another it was unlimited until the participant gave correct answers [14]. In two studies level of access was not stated [18, 20].

Outcome measures varied. Knowledge was the most common outcome (in ten trials: [16–25]). Attitudes and cognitions were reported in five trials [6, 16, 18, 23, 24]. Three trials reported behaviour outcomes, including procedural skills and time taken to respond to a patient emergency [6, 14, 15]. No trial reported all three categories of outcome. (Further detail is reported in Electronic Supplementary Material: Tab. S1).

### Risk of bias assessment

**Fig. 2:**
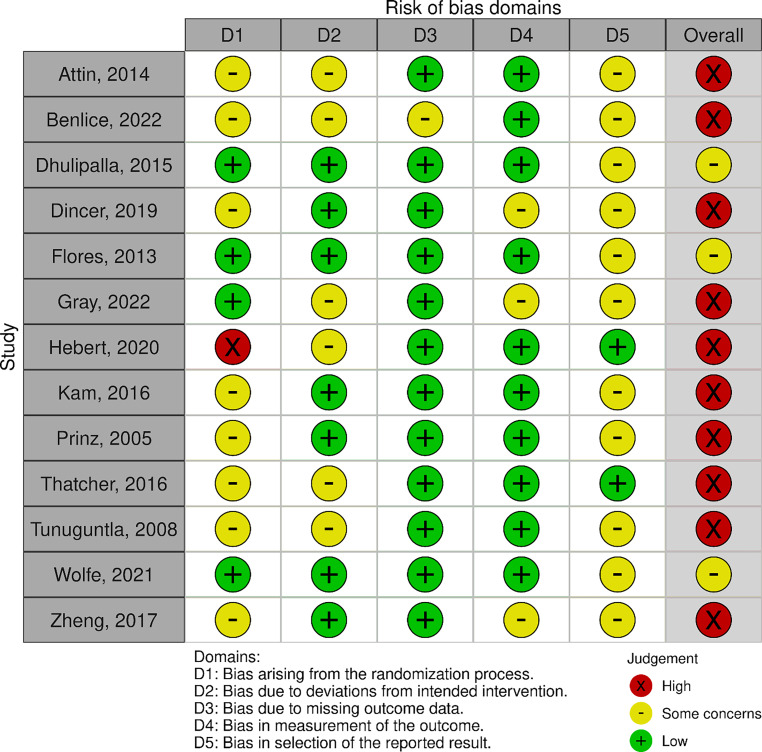
Risk of bias

Following the ROB2 algorithm, risk of bias was rated overall as ‘high’ in ten of the 13 trials (and as ‘some concerns’ in the remainder) (Fig. 2). Most trials had low risk of bias on two of the five domains (completeness of outcome data; selective reporting), which is a positive finding. However, risk of bias was present in most trials for the first domain (mostly in relation to a lack of allocation concealment, which could introduce confounding to the trial) and the fifth domain (mostly due to a lack of prior sample size calculation, without which null results are hard to interpret, or lack of protocol registration, which requires data analysis to be defined prior to data collection).

### *Outcomes (also see *Tab. S1*)*

#### Effects on knowledge

Knowledge was assessed in ten trials [16–25] and provision of an animation resulted in positive outcomes in eight of them. The animation was provided in addition to other information in five of these studies and resulted in increased student knowledge in four studies [16, 19, 22, 25]. When it was provided instead of another format (in eight studies), it produced better student knowledge when tested straight after intervention [17–21, 23, 25] and at later follow-up: at 1‑month post-intervention [21] and 4‑month post-intervention [24].

#### Effects on attitudes and cognitions

Attitudes and cognitions were assessed in five trials [6, 16, 18, 23, 24], and animation provision resulted in positive outcomes in three studies [6, 18, 23], no difference in one study [24], and worse outcomes in one study [16]. When the animation was provided instead of another format, it resulted in more positive evaluations from students in two studies [6, 23] and on satisfaction[18]. It also impacted positively on confidence in knowledge [23]. It did not impact on cognitive burden [24]. When provided in addition to another format [16], it resulted in less positive evaluations in three out of five reported measures and there was no difference between groups on the remaining two measures.

#### Effects on behaviours

Behaviours and skills were assessed in three trials [6, 14, 15] with animation provision resulting in positive outcomes in two of the studies [6, 14]. In one study it was provided in addition to another format and resulted in better performance scores and faster time to initiate resuscitation [14]. When provided as an alternative to other information, animations produced improved performance scores in one study [6] and had no effect in the other [15]. In one study animations did not impact on time to complete the clinical skill [6].

## Discussion

### Summary of findings

This systematic review of controlled effectiveness studies of video animations resulted in the inclusion and narrative reporting of 13 trials, of which 11 had been undertaken with student practitioners. There was substantial variation across studies in many aspects of the work, particularly the content and style of animations, outcome measures, and study populations; consequently, data pooling was not possible. The individual study results showed consistently positive effects of animations on knowledge, with two trials reporting longer-term improvements. Participants’ evaluations or preferences were mostly positive in favour of animations, although in one study the outcomes were more positive in the control group. Among the three trials that measured participants’ behaviours or skills, two studies reported more positive outcomes from animations. In all, the trials were mostly small and rated at risk of bias, reducing the certainty of findings.

### Strengths and limitations of the research

The systematic review involved a number of processes to increase rigour and reduce potential for bias: protocol registration, multiple database searching, use of entry criteria, inclusion of non-English language articles, citation searching, and dual decision-making on study inclusion, data extraction and quality appraisal.

The volume of included evidence is small, comprising just 13 studies in total and only two evaluating animations in qualified practitioner education. In total the 13 studies allocated just over 1000 participants. The quality of the studies was mixed: all but one used random allocation, although other study features could have introduced bias (such as a lack of concealment of allocation) or could not be assessed due to non-reporting. Only one study [25] reported a sample size calculation to indicate statistical power, although several were described as pilot or feasibility trials, in which a sample size calculation would not be necessary. However, the inclusion of several very small trials (the median sample size was 60) all reporting positive outcomes does raise the possibility of publication bias.

Only four of the 13 studies provided a link to the tested animation, although some study reports included still images from the animation, to indicate content and style. However, without being able to play the videos, a detailed evaluation of the content, tone, accessibility, or quality of the animations was not possible; it also inhibits the possibility of study replication or the progressive development of interventions, which are crucial elements of robust science.

### What this evidence adds

This is the first systematic review of the effectiveness of video animations within practitioner or student practitioner education. Although the evidence base is small, it indicates mostly positive effects on outcomes, including positive effects on knowledge, self-confidence and user evaluations. There is limited evidence for the benefits on skills or performance, although there was no evidence of poorer outcomes from animations. Only three trials assessed longer-term knowledge outcomes: in educational settings, this would be a more important indicator of intervention success. Furthermore, the animations in this review had been evaluated as discreet interventions (indeed an entry criterion of the review was that their effectiveness could be differentiated); consequently, their effectiveness within a larger package of multimedia educational material (whether delivered online or offline) was not evaluated. The included studies are all pragmatic, real world evaluations which is a strength; however, one disadvantage is that they did not include any process data (such as eye tracking or attention monitoring) which could indicate individual engagement with the animations and provide insight into reported benefits.

### Implications of the findings

Multimedia educational packages, including video films and video animations, have become common in education over the past two decades, although there is a view that their potential has not been realised [26, 27]. However, there remains a lack of large-scale and high-quality evidence on their effects, as well as on their optimal design and content. One concern is that animations may facilitate or even encourage surface level (not deep) learning, particularly when covering detailed topics and when animations are short. There are further concerns that the length of users’ attention to video may be highly limited meaning that, with more complex or detailed topics, their useful function may be restricted to providing an overview or introduction. This potential weakness was not evaluated in any of the included primary studies; indeed, the reliance on short-term measures of knowledge (or recall) in most studies could mask a lack of deeper or more conceptual learning. Animations may work best to convey procedures or mostly factual content, although this presumption would benefit from empirical evaluation. For example, in non-healthcare settings the relative benefits of animations over static pictures were greater when procedural knowledge was being taught [8] and when a more realistic animation style was being used. When used with patients, animations have shown a similar pattern to those reported in this review: mostly beneficial effects on knowledge, and mixed findings (and much less evidence) on attitudes, cognitions and behaviour [7].

The development of animations carries both financial and opportunity costs; furthermore, their provision may disadvantage those with lesser access to computers or slow internet speeds. However, animations can be dynamic and so have potential to demonstrate procedures or clinical skills in ways that other formats, including static images or video of real actors, may struggle to do. This systematic review provides some evidence for their effectiveness in practitioner education, particularly on knowledge in the shorter term.

There are several research implications generated by this systematic review. Animations were not always shown to be beneficial in the included studies, but there are several results that indicate promising effects: these need replication, particularly in bigger, more definitive trials. As in patient settings [7], this review found a lack of research for the effects on behaviour, and this warrants further investigation. Furthermore, it would be useful for studies to assess the relative effects of ‘representational’ and ‘decorative’ animation styles, which was found to be important in non-healthcare education [8]. Fine-grained research into users’ attention and eye-tracking may also help to indicate the ways that animations can have benefits.

There are also some implications for study design. Future trials would benefit from including: sample size calculations; concealment of allocation at recruitment, potentially using cluster allocation; and an adjustment for statistical multiplicity when required. Evaluated animations really should be available to research users: without doing that it is almost impossible to discern their quality or estimate the effects of mediators (and so understand why some animations are effective while others are not) [8]. However, concerns about student equity may discourage the use of randomised study designs in education, even when equipoise is agreed. However, the use of wait-list controls or Latin Square study designs may lessen these concerns when their use is possible.

The lack of controlled study evidence in qualified practitioners is particularly noteworthy because the evidence from student practitioners is not necessarily applicable (given differences in baseline knowledge, and likely differences in educational expectations and age). Finally, the current evidence base does not indicate whether animations work better as a complement to, or replacement for other forms of provision, and this important educational point needs clarification.

## Conclusions

Overall, the current trial-level evidence base for animations in healthcare education is small and it would be imprudent to recommend their routine use at this stage. However, some studies reported impressive levels of outcome improvements from animations (at least in the short-term), and no study indicated worse outcomes on knowledge or behaviour/skills. There look to be significant potential benefits of using animations in practitioner education, particularly for teaching factual content and clinical procedures.

## Supplementary Information

Figure 1. Medline search strategy

Table 1. Summary of included studies
