# Supplementary material for: The effectiveness of video animations in the education of healthcare practitioners and student practitioners: a systematic review of trials
Source: Perspect Med Educ. 2022 Dec 6;11(6):309–15. doi: 10.1007/s40037-022-00736-6 (PMC9743876; doi:10.1007/s40037-022-00736-6)
Supplement: Supplementary file 2 — Table 1. Summary of included studies [file 40037_2022_736_MOESM2_ESM.docx]

**Table 1. Summary of included studies**

| **Author, year; country** | **Study design** | **Aim of information or education;**  **Participants; Education level in the trial arms** | **Age Mean (SD); % Male** | **Total sample;**  **Intervention descriptor & sample size (I);**  **Control descriptor & sample size (C)** | **Animation type; length; delivery and times viewed; and link to animation (if available)** | **Results (Knowledge; Attitudes & Cognitions; Behaviours): Intervention vs Control** | **Results summary** |
| --- | --- | --- | --- | --- | --- | --- | --- |
| **Attin, 2014; USA** | RCT (individual allocation) | To increase nursing students’ knowledge and skills in CPR;  Nursing Students (year 3);  Similar (graduates: I 4/17; C 3/14). | 28.3 (7.5);  NR. | n=31;    I: Animation of three short scenarios of finding unresponsive patient, one incorrect procedure and two correct, including questions to check student understanding, followed by tutored group discussion, (n=17);  C: Tutored group discussion only, (n=14). | Intervention: cartoon animation;  Length NR;  Viewed on PC once if questions answered correctly, repeated views if answered incorrectly;  Not available to view. | **Knowledge:**  Not assessed;  **Attitudes & Cognitions:**  Not assessed;  **Behaviours:**  (i) Time to respond to cardiac arrest (median (range)): I= 7.6 seconds (1.7-13.8) vs C= 42.7 (16.0-101.0); p<.001  (ii) Followed correct CPR for safe care n/N: I= 17/17 vs C= 12/13; p-value NR. | **Behaviour: Favours animation.** |
| **Benlice, 2022; Turkey** | RCT (individual allocation) | To compare  the effectiveness of various educational tools including surgical  textbooks, 3D animation and cadaveric videos in laparoscopic rectal  surgery among board-certificated surgeons. | Not stated | n=168;  I: 3D animated diagram of laparoscopic rectal surgery, (n=42);  C1: Cadaveric video with actual cadaver, (n=42);  C2: Textbook chapter from Turkish colorectal society including drawings and photos, (n=42);  C3: animation + cadaveric video, n=42. | 3D animated video; 9 minutes 52 secs; participants asked to view it at least twice. | **Knowledge:**  I: Mean (SD): 14.4 (2.9), n-37.  C1: 15.4 (3.1), n=32.  C2: 12.2 (3.9), n=30.  C3: 16.2 (3.5), n=31.  Animation vs cadaver p>.05.  Animation vs textbook p<.05.  Animation + cadaver vs cadaver p>.05.  **Attitudes & Cognitions:**  Not assessed;  **Behaviours:**  Not assessed. | **Knowledge:**  **Favours animation over textbook.** |
| **Dhulipalla, 2015; India** | RCT (individual allocation) | To increase dental students’ knowledge of peridontal health and its management;  Dentistry Students (year 1);  NR | Age NR;  7.5%. | n=80;  I: 3D animation on oral health education (anatomy, aetiology, presenting conditions, prevention & treatment); (n=40);  C: video content as above but 2D;  (n=40). | Intervention: 3D animation;  4.25 minutes;  Watched once in classroom;  Not available to view. | **Knowledge (mean (SD) score out of 10):**  (i) post-intervention: I= 9.0 (1.6) vs C= 6.9 (1.6); p<.001;  (ii) 1 month post-intervention: I= 8.4 (1.2) vs C= 6.0 (1.4); p<.001.  **Attitudes & Cognitions:**  Not assessed;  **Behaviours:**  Not assessed. | **Knowledge: favours animation.** |
| **Dincer, 2019; Turkey** | RCT (individual allocation) | To increase nursing students’ knowledge and skills in care of patients with respiratory problems;  Nursing Students (year 2);  NR | 19.4 (0.9);  18.3%. | n=60;  I: 3D animation on communication with patient, respiratory assessment & symptoms, physical examination & Interpretation, plus tutor delivered slide presentation, (n=30);  C: Tutor-delivered slide presentation only, (n=30). | Intervention: 3D cartoon animation;    Length NR;  Watched once on computer;  Not available to view. | **Knowledge (mean % correct (SD)):**  (i) Post-test: I= 84.2 (8.3) vs C= 72.5 (13.6); p<.01.  (ii) Posttest–pretest increase: I= 15.5 (11.5) vs C= 2.3 (3.6); p-value NR. But increase in both arms p<.01.    **Attitudes & Cognitions:**  Not assessed;  **Behaviours:**  Not assessed. | **Knowledge: favours animation.** |
| **Flores, 2013; USA** | RCT (individual allocation) | To teach students to apply an ivy loop to lower jaw;  Medical Students (years 1 and 2);  NR. | NR;  NR. | n=32;  I: 3D animated procedure of lower jaw surgery, (n=16);    C: Textbook description of procedure, (n=16). | Intervention: 3D animated procedure (without sound narration);  Length NR;  Watched once on computer;  Available to view. Article also includes animation images. | **Knowledge:**  Not assessed;  **Attitudes & Cognitions (mean score out of 5):**  Assessment of education:  Stimulating: I= 4.4 vs C= 2.4; p<.001;  Interesting: I= 4.3 vs C= 2.7; p<.001;  Allowed one to learn: I= 4.8 vs C= 3.0; p<.001;  Clear: I= 4.9 vs C= 2.5; p<.001;  Effective: I= 4.9 vs C= 2.4; p<.001;  Would recommend to others: I= 4.9 vs C= 2.1; p<.001.    **Behaviours (mean score (SD)):**  (i) Arch Bar assessment scale: I= 18.8 (2.9) vs C= 13.0 (3.5); p<.001.  (ii) Time to complete technique: I =158 seconds (52) vs C= 194 secs (111); p=0.14. | **Attitudes & Cognitions: favours animation.**  **Behaviours: Skills, favours animation; Procedure time taken, no difference between animation and textbook.** |
| **Gray, 2022; Australia** | RCT (individual randomisation) | To test the effects of additional video animation for its effects on Multiple Choice Question test scores. | Not stated. Median age 30-39 in both arms. | N=38;  I: Animation of 3^rd^ stage of labour + traditional classroom teaching; n=20.  C: Traditional classroom teaching alone. n=18. | 3D animation; 8 minutes 13 secs; viewed once in classroom; available to view. | **Knowledge (mean; SD)**  Immediately post-teaching:  I: 22.3 (3.0) vs C: 18.7 (2.9); P<.001.  1 month later:  I: 21.2 (3.5) vs C: 19.4 (4.2); P=0.34.  **Attitudes & Cognitions:**  Not assessed.  **Behaviour:**  Not assessed. | **Knowledge favours animation** |
| **Hebert, 2020; Burkina Faso** | Non-randomised CT (allocation clustered by education group) | To increase healthcare workers’ knowledge of transmission of dengue fever and relevant research;  Nursing students (years 1 to 3);  NR | Age NR;  40.0%. | n=239;  I: Animation of dengue fever transmission; (n=81);  C1: ‘Journalistic’ style video; (n=79);  C2: ‘Dramatic’ style video; (n=79). | Intervention: cartoon animation;    4.38 minutes;  Watched once in classroom;  Not available to view. Article includes animation images. | **Knowledge (% correct (95% CI))** :  I: 59.5% (55.6-63.4) vs C1 52.9% (49.3-56.5) vs C2 61.2% (57.9-64.5);  I vs C1; p<0.029;  I vs C2; NS.  **Attitudes & Cognitions:**  Not assessed;  **Behaviours:**  Not assessed. | **Knowledge:**  **favours animation over journalistic style; No difference between animation and dramatic style.** |
| **Kam, 2016; Australia** | Crossover RCT  (individual allocation) | To increase qualified nurses’ knowledge of cystoscopy and stent insertion in renal colic;    Registered Nurses;  NR, similar years post-registration experience I: 10.8 years, C: 8.5 years, p=0.37. | 38.5 years;  7%. | n=72;  I: Cartoon animation; (n=39);  C: Spoken information including diagrams; (n=33). | Intervention: carton animation;  7.1 minutes;  Watched on tablet or laptop, frequency NR;  Not available to view. | Only data from first allocation in the trial have been included.  **Knowledge (mean correct out of 32 (95%CI)):**  I= 25.9 (25.2-26.6) vs C= 24.3 (23.5-25.1); p=.004;  **Attitudes & Cognitions (mean % (95%CI)):**  Satisfaction with information:  I= 29.5 (28.3-30.7) vs C= 26.5 (25.1-27.9); p=.003.  **Behaviours:**  Not assessed. | **Knowledge: favours animation.**  **Attitudes & Cognitions: favours animation.** |
| **Prinz, 2005, Austria** | RCT (allocation clustered by education group) | To teach students the relevant ‘topographical understanding’ related to eye surgery;  Medical students;  NR. | Age NR;  38.9%. | n=172;  I: 3D animated surgical procedure of cataract and glaucoma surgery, plus video showing the surgeon’s view (n=90)  C: Video showing the surgeon’s view, (n=82) | Intervention: 3D animated procedure;  10 minutes;  Watched once in lecture room;  Not available to view. Article includes animation images. | **Knowledge:**  (i) Topographical understanding (mean % correct): I= 75% vs C= 59%; p<.001.  (ii) Theoretical understanding: I= 72% vs C= 61% (NR); p<.001.  (iii) All topics: I= 12% higher than C (95% CI of difference 7 to 17); p<.0001.  (iv) Cataract topics: I= 14% higher than C (means NR) (95% of difference 7 to 21); p<.0001.  (v) Glaucoma topics: I= 11% higher than C (means NR) (95% CI of difference 5 to 17); p=.0002.  **Attitudes & Cognitions (mean score out of 4 (range)):**  (i) Satisfaction with learning aid: I= 1.2 (e 1-3) vs C= 1.3 (1-4); p=0.14.  (ii) Useful learning aid: I= 1.4 (1-3) vs C= 1.6 (1-4); p=.02.  (iii) Intelligibility for cataract surgery: I= 1.7 (1-3) vs C= 1.7 (1-3); p=0.69.  (iv) Intelligibility for glaucoma surgery: I= 1.6 (1-3) vs C= 1.8 (1-3); p=.03.  (v) Improvement of spatial ability: I= 1.4 (1-3) vs C= 1.7 (1-4); p=.01.  **Behaviours:**  Not assessed. | **Knowledge:**  **favours animation.**  **Attitudes & Cognitions: favours control on 3 items; no difference between arms on 2 items.** |
| **Thatcher, 2016; USA** | Crossover RCT  (individual allocation) | To increase students’ understanding of DNA and its replication;  Osteopathy Students;  NR. | NR;  NR. | n=22;  I: Animation, (n=12);  C: Textbook, (n=10). | Intervention: animated models;  Length NR;  Watched on CD-ROM on PC;  Frequency NR;  Not available to view. Article includes animation images. | Only knowledge data from first allocation in the trial have been included.  **Knowledge:** (% correct (SD)):  I= 96.3 (4.0) vs C= 74.0 (12.0); p=.01.  **Attitudes & Cognitions:**  Not assessed.  **Behaviours:**  Not assessed. | **Knowledge:**  **favours animation.** |
| **Tunuguntla, 2008; USA** | RCT (individual allocation) | To increase students’ knowledge of home safety aspects in Elderly Care;  Medical Students (year 1);  Similar. | Age NR;  56%. | n=50;  I: Animation, (n=23);  C: Static graphics, (n=27). | Intervention: cartoon animation;  Length NR;  Watched on PC,  unlimited access;  Not available to view. | **Knowledge (mean (SD)):**  Home safety knowledge (4 months after intervention):  I= 93.0 (7.2) vs C= 90.0 (8.7); p= 0.15.  **Attitudes & Cognitions (mean (SD)):**  Cognitive burden (higher score = more demand):  I= 1.6 (0.6) vs C= 2.1 (1.1); p=0.13.  **Behaviours:**  Not assessed. | **Knowledge: no difference between arms.**  **Attitudes & cognitions:**  **No difference between arms**. |
| **Wolfe, 2021; USA** | RCT (individual allocation) | To increase students’ knowledge of facial transplantation and relevant head and neck anatomy;  Medical Students (years 3 & 4);    Similar. | I: 25.8 years (SD 1.5); C: 26.1 (1.8);  44.4% | n-36;  I: 3D animation of procedure and anatomy, (n=19);  C: Text version of narration plus still images, (n=17). | Intervention: 3D animation;  Length NR;  Watched once, delivery NR;  Available to view (behind paywall). Article includes animation images. | **Knowledge (mean (SD)):**  I= 11.3 (1.5) vs C= 9.6 (2.6); p=0.029.  **Attitudes & Cognitions (mean out of 6 ):**  Confidence in knowledge of transplantation: I= 3.5 vs C= 3.2; p=0.002.  Confidence in knowledge of head & neck anatomy: I= 3.6 vs C= 3.4; p=0.073.  Satisfaction with resource:  Stimulating I= 4.8 vs C= 2.8 ; p<.001.  Interesting I= 4.8 vs C= 3.0 ; p<.001.  Allowed better learning I= 5.0 vs 4.2 ; p<.001.  Clear: I= 5.0 vs 4.0 ; p<.001.  Effective: I= 5.4 vs 4.0 ; p<.001.  Would recommend to others: I= 5.4 vs 3.4; p<.001.  **Behaviours:**  Not assessed. | **Knowledge: favours animation.**  **Attitudes & Cognitions: Self-confidence favours animation (both items).**  **Satisfaction all six items favour animation**. |
| **Zheng, 2017; USA** | RCT (individual allocation) | To increase students’ skills in managing 3 emergency situations (drowning, choking, bone fracture);  Nursing Students (year 1);    Similar. | Age NR;  NR. | n=68;  I: Cartoon animation, (n=34);  C: Spoken lecture and demonstration, (n=34). | Intervention: Cartoon animation;  5 minutes;  Watched 4 times in classroom;    Not available to view. Article includes animation images. | **Knowledge**:  Not assessed.  **Attitudes & cognitions:**  Not assessed;  **Behaviours (mean % increase pre-post):**  Procedure skills : I= 35% vs C= 37%; NS. | **Behaviour: no difference between arms.** |

NS= Not statistically significant (p>.05); SD = Standard Deviation; NR= Not reported; I= Intervention; C=Control; vs = versus; RCT = randomised control trial; CT = controlled trial; 3D = three-dimensional; 2D = two-dimensional.
